# COVID-19 and Disenfranchised Grief

**DOI:** 10.3389/fpsyt.2021.638874

**Published:** 2021-02-12

**Authors:** Sara Albuquerque, Ana Margarida Teixeira, José Carlos Rocha

**Affiliations:** ^1^Center for Research in Neuropsychology and Cognitive and Behavioral Intervention, Faculty of Psychology and Educational Sciences, University of Coimbra, Coimbra, Portugal; ^2^Clinical, Research and Training Center “PIN—em todas as fases da vida”, Lisbon, Portugal; ^3^HEI-Lab, Universidade Lusófona, Lisbon, Portugal; ^4^Behavioral and Social Sciences Department, Cooperativa de Ensino Superior Politécnico e Universitário (CESPU), Porto, Portugal; ^5^Center for Grief and Trauma Psychology, Centro de Psicologia do Trauma e do Luto (CPTL), Porto, Portugal

**Keywords:** death, grief, bereavement, disenfranchised grief, COVID-19

## Introduction

Although death is an inherent part of life, for many it is a terrifying event, which awareness is often to be avoided at all costs (1). However, with the daily updates of COVID-19 cases and deaths, the confrontation with human mortality and physical fragility is unavoidable. Over 100 million confirmed cases and over 2 million confirmed deaths worldwide have been recorded (2). The COVID-19 pandemic is a health crisis unprecedented in contemporary history.

Furthermore, an estimate of 9 bereaved family members results from each COVID-19 death (3). Recent evidence indicates that, due to the circumstances in which deaths in the COVID era occur—unexpected and shocking deaths, social distancing, restrictions in visits in healthcare facilities and in funerals—another epidemic is on the rise: prolonged grief disorder (PGD) [e.g., (4)]. PGD is characterized by persistent and pervasive longing for, or preoccupation with the lost one, as well as severe emotional pain (such as, guilt, anger, or sadness), difficulty accepting the death, emotional numbness, a sense that a part of them has been lost, an inability to experience positive mood and difficulty participating in social activities (International Classification of Diseases-11, (5)).

With each death that occurs, there are loved ones who will be deeply impacted by the loss, particularly at a time when, due to sanitary restrictions, they may experience limited autonomy, and resourcefulness when coping with their grief. The awareness of these limitations heighten the risk of grievers experiencing their grief as disenfranchised to a degree.

Kenneth Doka first formally introduced the notion of disenfranchised grief in 1989 and defined it as the process in which the loss is felt as not being “openly acknowledged, socially validated, or publicly mourned” (1989, p. xv). This experience of grief might pose difficulties in terms of emotional processing and expression, as one may not recognize his/her right to grieve, and in terms of social support, by diminishing the opportunity to freely express their emotions, and to obtain expressions of compassion and support (6).

Given the challenges that the disenfranchisement of grief might add to the bereavement experience, it is important to reflect on the risk for this experience in the context of the COVID-19 circumstances. In this opinion paper we aim to frame this in light of the felt limitations in autonomy and resourcefulness in the COVID-19 bereaved, either imposed externally, or internally (self-disenfranchisement).

## Disenfranchisement Imposed Externally

Doka (6) suggests that which losses and which relationships have the legitimacy of being grieved is defined by each society's grieving norms. When a loss does not accommodate these guidelines, the resultant grief remains unrecognized and undervalued and a person may feel as his/her “right to grieve” has been denied. So it seems that there are bereavements that are not socially acceptable, in which its grieving is complex and triggers secondary variables like the disenfranchised grief. According to Corr (7) an aspect that contributes to the grieving process being disenfranchised is the devaluing of public mourning rituals. Arguably, restrictions are needed to contain the spread; however, there are concerns about how current restrictions in end of life care and funeral rites may compromise the salutary grieving process and amplify the risk for disenfranchised grief.

The usual rituals, customs and interactions that occur in the context of end of life and after a death are another casualty of the virus. During end of life, in order to conform with the guidelines for preventing the spread of the disease, patients are often faced with separation from their loved ones reporting high levels of loneliness, uncertainty and despair. In the same way, it could increase feelings of neglect and dehumanized treatment (8).

In addition, Bromberg (9) identifies therapeutic functions in the funeral rituals: (1) they assist family members and friends in recognizing and confronting the reality of the loss; (2) they offer room for introspection on the death as a process integrated into life; (3) they foster the awareness and assimilation of the grief process. These therapeutic functions may be deeply compromised currently as families are deprived from access to social support and usual burial and funeral rituals.

Funeral rituals have been taking place with a reduced number of family members present, leaving the online mode as the only option for many people. These virtual interactions don't replace usual in person interactions and rituals. Not only did they previously include both immediate and extended family members and friends but allowed for physical connection and touch—a pat in the back, a stroke, a hug (10). Sharing their pain with other bereaved people, and having a place of memorialization, may foster emotion expression and meaning-making for bereaved people (11, 12). Therefore, bereaved people may find the shortage or minimalistic nature of funerary rituals painful (13). Also challenging is the perception that their loved one didn't receive the funerary ritual they deserved, not being able to say farewell to the deceased and not being able to visit the grave afterwards (14).

Likewise, feeling disallowed and unsupported to openly express and manage their grief may contribute to disenfranchisement (15). We are facing times that compromise the normal and necessary social support usually available during the illness and after death. Touch is a basic human need, especially in grief, and can help the person in their coping process after a loss (16). It could help to ground in the present moment (here and now), to have someone, something to hold on to.

Finally, the increasing accumulation of deaths could impede acknowledgment of each individual's grief. The deaths framed as statistic also leaves the bereaved individuals feeling that the pain of their loss in undervalued. In addition, not only can grief be overlooked by society but patients and their families can also experience social stigma regarding COVID-19 losses (17).

## Self-Disenfranchisement

Expanding on Doka's original work on disenfranchised grief, Kauffman (18) proposed the concept of self-disenfranchisement, which occurs when individuals have difficulty in acknowledging their own grief as being legitimate. One proposed associated emotion of these situations is guilt, which is especially present in situations of unexpected or sudden loss (19). In the present pandemic context, bereaved people have scarce opportunities to prepare for their loss as, after contracting the virus, it can only take weeks or even days for the person to die. Literature has shown that sudden deaths in the context of intensive care are associated with more mental health problems (20), and highlight the particular impact of the lack of information and the consequent intrusive constant doubts (21). Examples of these doubts in the present context may refer to whether the person has suffered or if they have received appropriate care (associated with the perception of resource scarcity). They may also wonder why they were spared and whether they had a part in the person getting infected (8). Also, the bereaved may feel guilty for not being able to be present at the time of death; for not having reaffirmed their love, providing comfort as they wished to or saying goodbye (22). An experience of guilt-shame at their impotence may therefore be magnified in the current pandemic context (23).

On the other hand, self-disenfranchisement may be related to not having the emotional safeness conditions and coping resources in order to fully connect with their pain and grieving process. One aspect that may contribute to this is the current rise in pandemic-related anxiety and depression levels. It may deplete the emotional resources needed to grieve the loss of the person, on its own, or due to the risk of triggering previous mental illness issues (24). Also, the overload concept of the well-established Dual Process Model (25) may be particularly representative of the current grieving context. These authors argue that apart from the necessary oscillation between loss-oriented coping (confronting and handling feelings of grief and loss) and restoration-oriented coping (stressors of daily life that result from the death) it is important to evaluate the existence of more loss or restoration stressors than the person feels capable of handling. It is our view that the COVID-19 pandemic poses serious risks of overload of restoration stressors. First of all, simultaneously to the already arduous process of grieving, individuals are experiencing varying degrees of personal, economic and social losses (26). Also, as people are being deprived of usual social, recreational, and occupational activities, there are less opportunities for taking time off from the overwhelming emotional experience of grief. Likewise, without face-to-face meetings, after-death practicalities (e.g., sort out phone contracts in the deceased person's name, bank accounts, liaise with funeral directors) may be more difficult to some, especially those with less technology resources, paving the way to difficulties in accepting the loss. Finally, access to support services and social support is much harder. In general, overall needs for grieving and bereavement are largely unattended.

## Conclusion and Clinical Implications

Despite acknowledging the importance of future research investing at primary empirical data, in this opinion paper we aimed at offering a preliminary and exploratory view of the risk for disenfranchisement in grief and bereavement in the current pandemic context.

Therefore, in this context, access to evidence-based bereavement psychological interventions is critical. The existing gaps in the area of mental health can contribute to the lack of investment among the bereaved. Therefore, in order to better prepare for managing grief-related mental health issues, we must first increase general public's literacy around mental health and grief (grief-informed communities), combating stigma and discrimination (27).

For this purpose, we need a public health approach aimed at restoring the normal social and community support systems. It is also important to be aware of risk factors in order to intervene earlier and effectively in the mental health issues and in the grief process. Simultaneously, it could be relevant to build intervention protocols that involve screening and mental health risk assessment for those with greater risk factors for grief complexities.

In addition, specific recommendations regarding coping with social restrictions in funerals, access to social support, and in regards to promoting more supportive social perceptions for bereaved people are offered in Table 1. Recommendations to address feelings of impotence, guilt and shame and lack of emotional and coping resources are offered in Table 2.

**Table 1:** Recommendations regarding disenfranchisement imposed externally to promote healthy COVID-19 related grieving.

**Changes due to COVID-19**	**Impact**	**Disenfranchisement imposed externally: Recommendations**
Limitations to funerals and/or burials	Not being able to say goodbye Unfinished business Survivor guilt Loss of social support	Discuss about desired ritual or spiritual practices and funeral/memorial Helping families to memorialize or remember the deceased in alternative and innovative ways Collective rituals (e.g., express feelings through phone calls, letters, text, and audio messages) Virtual religious celebrations such as cults, masses, funerals and online memorials in which family members, friends, and others can express their condolences and share thoughts about the loved one
Limitations in visits and in end of life care		Assisting families in working through the information and decision in the dying process Validating their emotions and gaining awareness of potential salutary coping skills according to their values and beliefs Helping brainstorm creative strategies for maintaining closeness and communication using technology Psychoeducation on the grief process, particularly regarding risk factors, and warning signs
Limitations in access to social support	Loss of social/physical connections and support	Assisting families in accessing social support networks practices and funeral/memorial plans Help in accessing resources to help them plan for practical needs after the death Provide access to additional grief support Enhancing the importance of self-care Promoting contact with other people going through the same experience in order to foster reciprocity and reduce isolation Highlighting the need of bereaved people in terms of feeling listened to and in everyday tasks
Negative social perceptions	Stigma Feelings that the loss is not acknowledged by society	Publicly organized commemoration activities to foster collective grief and a sense of belonging Provide a minute of silence Taking actions to improve de grief literacy in the general population

**Table 2:** Recommendations regarding self-disenfranchisement to promote healthy COVID-19 related grieving.

**Changes due to COVID-19**	**Impact**	**Self-disenfranchisement: Recommendations**
Unexpected or sudden loss	Feelings of impotence, guilt, and shame	Validating their emotions and give space to their expression Using memory to create compassionate feelings (e.g., recall the feelings when one has experienced the kindness of others) Help in building compassionate approaches to cope with the emotions (e.g., focus the attention in the present moment rather than becoming distracted by “what if's?”) and rumination processes Keep in mind that things and feelings change Imagine oneself as a compassionate person speaking to a friend Encourage the integration of the idea “I did the best I could, with the knowledge I had.” Reach out to others and see if help is available Understanding what one can and can't control
Depletion of emotional and coping resources	Lack of emotional safeness conditions	Psychoeducation on the risk factors and warning signs of common mental health disorders (e.g., anxiety and depression) Encourage people to ask for professional help Improving access to psychological interventions, support services, and social support Promoting strategies of self-care and values and meaningful future plans Monitoring the oscillation between loss-oriented coping (confronting and handling feelings of grief and loss) and restoration-oriented coping (focused on secondary stressors)

COVID-19 related deaths are in multiple ways lonely and dehumanized processes for patients and families. Limitations in self-efficacy, choice, and control not only changed the landscape of grief and grieving but pose a significant risk and added burden in the already arduous and painful grieving experience.

## Author Contributions

SA and AT contributed to the development of the idea, the literature research, and the writing of the manuscript. JR provided critical feedback.

## Conflict of Interest

The authors declare that the research was conducted in the absence of any commercial or financial relationships that could be construed as a potential conflict of interest.
